# Dairy food intake is not associated with spinal trabecular bone score in men and women: the Framingham Osteoporosis Study

**DOI:** 10.1186/s12937-022-00781-1

**Published:** 2022-05-10

**Authors:** Courtney L. Millar, Douglas P. Kiel, Marian T. Hannan, Shivani Sahni

**Affiliations:** Department of Medicine, Hinda and Arthur Marcus Institute for Aging Research, Hebrew SeniorLife, Beth Israel Deaconess Medical Center and Harvard Medical School, 1200 Centre Street, Boston, MA 02131 USA

**Keywords:** Trabecular bone score, Dairy food, Bone, Older adults

## Abstract

**Background:**

Previous studies reported that dairy foods are associated with higher areal bone mineral density (BMD) in older adults. However, data on bone texture are lacking. We determined the association of dairy food intake (milk, yogurt, cheese, milk + yogurt and milk + yogurt + cheese) with spinal trabecular bone score (TBS).

**Methods:**

In this cross-sectional study, a validated semi-quantitative food frequency questionnaire was used to assess dairy food intake (servings/wk). TBS, an analysis of bone texture, was calculated from dual energy X-ray absorptiometry (DXA) scans. Sex-specific multivariable linear regression was used to estimate the association of dairy food intake (energy adjusted via residual methods) with each bone measure adjusting for covariates.

**Results:**

Mean age of 4,740 participants was 49 (SD: 13) years and mean milk + yogurt + cheese intake was 10.1 (SD: 8.4) servings/week in men and 10.9 (SD: 8.0) servings/week in women. There were no associations between dairy food intake and spinal TBS in adjusted models.

**Conclusions:**

In this cohort of primarily healthy adults, dairy intake was not associated with bone texture.

## Introduction

Osteoporosis is a major public health concern for nearly 54 million older Americans that are at risk of osteoporosis and related fractures [1]. It is estimated that more than 3 million osteoporosis-related fractures will contribute to approximately $25 billion dollars in direct medical costs by the year 2025 [2]. Therefore, there is an urgent need to prevent and treat osteoporosis. Calcium and vitamin D are the primary nutrients considered to prevent osteoporosis [3] and the U.S. Dietary Guidelines for Americans have included recommendations for dairy consumption, largely based on meeting recommendations for calcium intake for bone health in older adults [4].

Dairy foods are good sources of calcium, vitamin D (in fortified dairy foods), protein, and magnesium, nutrients that have been related to bone health [5]. Our previous research [6], and that of others [7–11], has suggested a positive link between milk intake and bone mineral density (BMD). A recent systematic review reported that daily intake of low- or non-fat dairy products as part of a healthy habitual dietary pattern may be associated with improved BMD of the total body and at some bone sites [12]. Yet, inconsistent findings of dairy intake and bone measures in recent studies [13, 14] has led to considerable controversy surrounding potential benefits of dairy foods for bone health. Previous studies on this topic have largely associated dairy food intake with dual energy x-ray absorptiometry (DXA) derived areal bone mineral density (aBMD), which can be confounded by individual differences in bone size and it does not provide information about bone microstructure, a key determinant of bone strength.

More advanced, novel imaging (e.g. bone microarchitecture analysis), use of existing images to generate measures associated with fracture risk beyond aBMD as well as common fracture risk tools like FRAX are now available. Yet, these methods have not been used by many nutrition studies. Protein intake specifically from dairy has been favourably associated with bone microarchitecture in older men [15] and post-menopausal women [16]. In the Geneva Retirees Cohort, consumption of fermented dairy products [17], reduced the peripheral cortical bone loss at distal radius in post-menopausal women. While informative for research, bone microarchitecture analyses are expensive and laborious, which may limit their use for routine clinical evaluations of bone health. Therefore, another novel method for assessing bone texture and fracture risk at the lumbar spine has been developed termed trabecular bone score (TBS). TBS is a less expensive evaluation of bone texture [18] that can be estimated from lumbar spine DXA images and has been correlated with measures of bone microarchitecture [19]. Only one cross-sectional study has reported a positive association of dairy food intake with TBS in community dwelling older Japanese men who likely have different intake of dairy foods than Americans of European ancestry [20]. Thus, the aim of this cross-sectional study was to determine the association of dairy food intake with TBS of the spine in men and women from the Framingham Heart Study (FHS). Our hypothesis was that higher intake of all dairy foods would be associated with higher TBS in men and women.

## Materials and methods

### Participants and study design

**Fig. 1:**
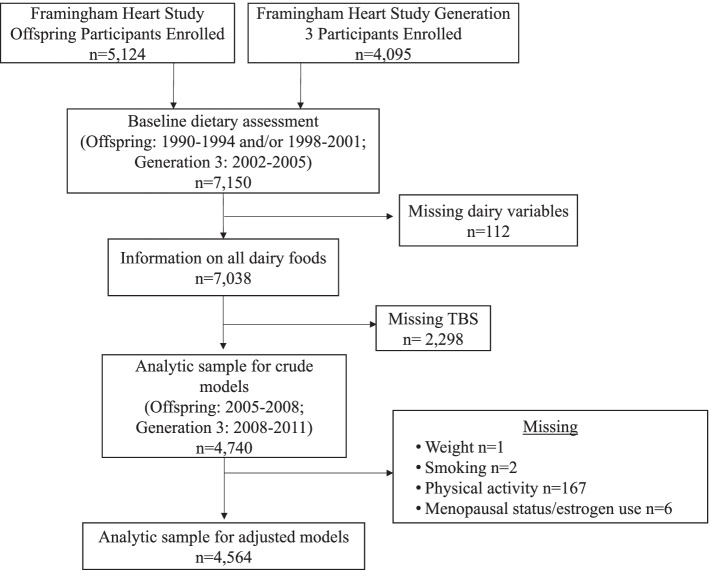
Flow chart of Framingham Heart Study (FHS) Offspring and Generation 3 (Gen3) cohort participants

To determine the association between dairy food intake and TBS, we included participants from the FHS Offspring and Generation 3 cohorts (Offspring and Gen3, respectively) with information on dairy foods from an FFQ, TBS assessment, and relevant covariates. The Original cohort participants were enrolled to assess cardiovascular risk factors at approximately four year intervals. The adult children and their spouses of the population-based FHS Original cohort (i.e., the Offspring Cohort) were enrolled in 1971 to 1975 (*n* = 5,124; age range 5–70 y at enrollment) [21]. The children of the Offspring cohort (i.e., Gen3) were enrolled in 2002–2005 (*n* = 4,095). There were 3,350 Offspring and 3,800 Gen3 participants (total *n* = 7,150) with validated dietary assessment at baseline examination (Offspring: 1990–1994 and/or 1998–2001; Gen3: 2002–2005) who were eligible for this study. Of the 7,150 participants, 112 were excluded due to missing information on intake of dairy foods and 2,298 were excluded due to missing TBS assessment at the follow-up examination (Offspring: 2005–2008; Gen3: 2008–2011). There were no exclusions for participants with missing or invalid FFQ [energy intake < 2.51 or > 16.74 MJ (< 600 or > 4,000 kcal/d)]. After excluding those with missing TBS assessment, 4,740 participants were included in the crude models. For fully adjusted models, we further excluded participants with missing information on covariates at the baseline examination (weight, *n* = 1; smoking, *n* = 2; physical activity, *n* = 167; menopausal status/estrogen use, *n* = 6; Fig. 1). The final analytic sample included 4,564 individuals.

All FHS participants provided informed consent. This study was approved by the Institutional Review Board at the Marcus Institute for Aging Research, Hebrew SeniorLife.

### Dietary assessment

A validated [22, 23] semi-quantitative Harvard FFQ [24] was used to estimated usual dietary intake for each participant at the index examination. Participants were mailed the FFQ and were asked to bring the completed questionnaire to their scheduled visit, where they were reviewed by the clinical staff. The FFQ included questions on frequency with a standard serving size and 9 categories ranging from never or < 1 serving/mo. to > 6 servings/d. Cumulative dairy food intake was the primary exposure for all analyses and was assessed using the food list section of the FFQ averaging across two examinations, if available in the Offspring cohort. Gen3 participants had one FFQ available and these data were used for analyses. The serving size for each dairy food is as follows: skim/low or fat/whole milk (8 oz. glass), ice milk (1/2 cup), cottage or ricotta cheese (1/2 cup), other cheese (1 slice or 1 oz. serving), and yogurt (1 cup). Milk intake was calculated as the sum of intake of skim milk, low-fat milk, whole milk, and ice milk. Cheese intake was calculated as the sum of intake of cottage/ricotta cheese and other cheeses (including cheeses from mixed dishes such as pizza or lasagna). Yogurt intake was estimated in servings per week. Fluid dairy intake was defined as the sum of milk and yogurt intake in servings per week and total dairy intake was defined as the sum of intake of milk, yogurt, and cheese in servings per week. We did not include cream intake in our variables since cream intake (e.g., cream, sour cream, ice cream, cream cheese, and butter) is not considered “Dairy” per the United States Department of Agriculture Food Groups [25].

### Spinal Trabecular Bone Score (TBS)

In the FHS Offspring and Gen3 cohorts, BMD of the spine (mean BMD from L2 to L4 in g/cm^2^) was scanned with the use of a GE Lunar Prodigy fan-beam densitometer (GE Healthcare Inc.) in 2005–2008 (Offspring) and 2008–2011 (Gen3). TBS is a measure of bone texture that can be used in the clinical setting [18] along with a DXA scan. TBS was measured from previously acquired DXA scans of the spine using the TBS iNsight® software (version 4, Medimaps Group, Geneva, Switzerland) as previously described [26]. In brief, TBS was evaluated based on the gray-level analysis of spinal DXA images as the slope at the origin of the log–log representation of the experimental variogram. This updated version of the software accounts for soft tissue thickness directly and improves fracture prediction in both men and women [26]. Average TBS and BMD from the spine’s lumbar vertebrae 2–4 was calculated for each individual before analysis.

### Covariates

Covariates were taken from the examination closest to the time when dietary assessment was conducted and included age (years), sex, height (inches), weight (pounds), physical activity, current smoking, total energy (kcal/d), multivitamin supplement use (yes/ no), calcium or vitamin D supplement use (yes/no), and for women menopause status/estrogen use. Since loss of estrogenic hormones in post-menopausal women is an important modulator of bone density [27], female participants were classified as 1. pre-menopausal, 2. post-menopausal and no estrogen use, or 3. post-menopausal with estrogen use. A variable for cohort was also considered as a covariate.

Height (inches) was measured while participants were without shoes to the nearest 0.25 inches (0.64 cm) using a stadiometer and converted into meters. Weight (pounds), in light clothing was measured with a standardized balance-beam scale. Physical activity was assessed with the use of a structured questionnaire to indicate the number of hours spent performing 5 levels of activity (i.e., being asleep, being sedentary, and performing light, moderate, and heavy activity). The responses contributed to a weighted sum (i.e., the physical activity index, PAI) with scores ranging from 24 (representing 24 h of sleeping) to 120 (representing 24 h of constant strenuous activity) [28]. Total energy (kcal/d), multivitamin supplement use (yes/no), and calcium or vitamin D supplement use (yes/no) were assessed using the FFQ’s food-list section. The smoking status of the participants was assessed via questionnaire as current cigarette smoker (smoked regularly in the past year), former smoker, or never smoker. Former and never smokers were combined into the group “non-current smokers.” Information on current estrogen use and menopause status (cessation of menses for at least 1 year) was obtained from self-reports on a questionnaire and verified by a medical chart review.

### Statistical analysis

Baseline characteristics are presented by sex. Categorical variables are presented as percentages and continuous variables are presented as mean and standard deviation (SD). A previous study has reported that lifestyle factors associated with TBS are not uniform across the sexes [29]; therefore, for this study we primarily conducted sex-specific analyses. If the associations in men and women were similar then we performed sex-combined analyses. Pearson’s correlation was calculated for L2-L4 lumbar spine BMD and TBS.

The primary exposure variables were milk, yogurt, cheese, milk + yogurt, and milk + yogurt + cheese intake in servings per week. Separate analyses were performed for each dairy food variable. Dairy food variables were adjusted for total energy intake by the residual method [30] and modeled as continuous variables. Multivariable linear regression was used to calculate regression coefficients (β) estimating the difference in bone measures associated with a 1-unit difference in dairy food intake (servings per week).

Initially, crude linear regression models were examined. Subsequent models were adjusted for age, height, weight, energy intake, current smoking, supplement use of calcium, vitamin D and multivitamins, physical activity, and cohort type (Offspring or Gen3). Models for women were further adjusted for the combined variable for menopause status and estrogen use (pre-menopausal women, post-menopausal women with no estrogen use, or post-menopausal women with estrogen use). To account for wide age range of the study participants, secondary analyses were performed such that final models were stratified by age (≤ 60 years versus > 60 years), and then further stratified by sex. All analyses were performed using statistical software program SAS (version 9.4, SAS Institute Inc.). A *p*-value < 0.05 was considered statistically significant.

## Results

### Baseline characteristics

Mean age of participants (*n* = 4,740) was 49 years (SD 13; range 19–88) for men and 49 years (SD 13; range 19–83) for women (Table 1). More than half of the participants were from the Gen3 cohort (> 57% in men and > 55.9% in women). Nearly 8% of the men and 42% of women used calcium supplements. Only 41% of men reported the use of multivitamins compared to 57% of women users. Milk was the most commonly consumed dairy food for both men [mean (SD): 5.8 (6.8) serv/wk] and women [mean (SD): 6.0 (6.4) serv/wk]. In this cohort, spinal TBS was strongly correlated with L2-4 spine BMD (*r* = 0.45, *p* < 0.001).

**Table 1:** Baseline characteristics of men and women from the Framingham Heart Study Offspring and Generation 3 cohorts

Descriptive variables^1^	Men (*n* = 2,158)	Women (*n* = 2,582)
Age, y	49 ± 13	49 ± 13
Offspring cohort, n (%)	928 (43.0)	1,139 (44.1)
BMI, kg/m^2^	28.1 ± 4.4	26.5 ± 5.9
Weight, lbs	193.6 ± 32.3	155.4 ± 35.1
Physical activity index z-score	0.2 ± 1.1	-0.2 ± 0.9
Menopause status and estrogen use Pre-menopausal, n (%) Post-menopausal, no estrogen use, n (%) Post-menopausal, estrogen use, n (%)	-	1,267 (49.2) 874 (34.0) 434 (16.9)
Current smokers, n (%)	269 (12.5)	314 (12.2)
Calcium supplement user, n (%)	166 (7.7)	1,085 (42.0)
Multivitamin supplement user, n (%)	890 (41.2)	1,471 (57.0)
Vitamin D supplement user, n (%)	34 (1.8)	144 (5.6)
Dairy intake (servings/wk)		
Milk	5.8 ± 6.8	6.0 ± 6.4
Yogurt	0.8 ± 1.7	1.5 ± 2.1
Cheese	3.5 ± 4.2	3.4 ± 3.7
Milk + yogurt	6.7 ± 7.1	7.5 ± 6.8
Milk + yogurt + cheese	10.1 ± 8.4	10.9 ± 8.0
Other dietary intakes		
Energy, kcal/d	2,104 ± 666	1,843 ± 577
Total calcium, mg/d	885 ± 428	1,110 ± 530
Dietary calcium, mg/d	824 ± 390	825 ± 390
Total vitamin D, IU/d	356 ± 259	417 ± 281
Dietary vitamin D, IU/d	222 ± 140	216 ± 147
L2-4 spine BMD	1.32 ± 0.02	1.20 ± 0.19
Trabecular bone score	1.42 ± 0.09	1.38 ± 0.10

^1^ Presented as mean ± standard deviation or n (%) for all such values

BMD, bone mineral density; BMI, body mass index

### Association of dairy food intake and TBS

In sex-stratified analyses, in men and women 1 serving higher cheese intake was significantly associated with 0.002 (SD: 0.0005, men) to 0.006 (SD: 0.0006, women) units higher TBS in the crude models (P range: < 0.01 each for men and women respectively, Table 2). Similarly, 1 serving higher milk + yogurt + cheese intake was significantly associated with 0.0006 (SD: 0.0002, men) to 0.0011 (SD: 0.0003, women) units higher TBS in the crude models (P range: 0.01 each for men and women respectively). However, after adjusting for relevant covariates the magnitude of the associations reduced and the associations were no longer significant (P range: 0.50–0.72, Table 2). Given that the direction of associations was similar for men and women, sex-combined analyses were performed which showed that dairy food intake was not associated with TBS after adjusting for relevant confounders (P range: 0.53–0.98, Table 3).

**Table 2:** Association of dairy food intake with trabecular bone score in men and women from the Framingham Heart Study Offspring and Generation 3 cohorts

Dairy Foods (servings/wk)	Crude Model						Adjusted Models^1^					
	**Males (*n* = 2,158)**			**Females (*n* = 2,582)**			**Males (*n* = 2,082)**			**Females (*n* = 2,482)**		
	**beta**	**SE**	***P-value***	**beta**	**SE**	***P-value***	**beta**	**SE**	***P-value***	**beta**	**SE**	***P-value***
Milk	-0.0003	0.0003	0.26	-0.0007	0.0003	0.04	-0.0001	0.0003	0.97	0.0001	0.0003	0.62
Yogurt	0.0017	0.0011	0.12	-0.0005	0.0010	0.62	0.0004	0.0010	0.71	0.0002	0.0008	0.85
Cheese	0.0024	0.0005	** < 0.01**	0.0061	0.0006	** < 0.01**	0.0002	0.0005	0.66	0.0002	0.0005	0.72
Milk + Yogurt	-0.0001	0.0003	0.90	-0.0006	0.0003	0.07	0.0001	0.0003	0.80	0.0001	0.0003	0.58
Milk + Yogurt + Cheese	0.0006	0.0002	**0.01**	0.0011	0.0003	** < 0.01**	0.0001	0.0002	0.62	0.0002	0.0002	0.50

^**1**^Models adjusted for age, cohort, height, weight, current smoking, energy intake, calcium supplement use, vitamin D supplement use, physical activity, multivitamin use, and menopause status/estrogen use (in women alone)

*SE* Standard error

**Table 3:** Association of dairy food intake with trabecular bone score in the combined sample of men and women and stratified by age from the Framingham Heart Study Offspring and Generation 3 cohorts

Dairy Foods (serv/wk)	All participants (*n* = 4,564)			Age < 60 years (*n* = 3,607)			Age ≥ 60 years (*n* = 957)		
	**beta**^**1,2**^	**SE**	***P*-value**	**beta**^**1**^	**SE**	***P*-value**	**beta**^**1**^	**SE**	***P*-value**
Milk	0.0001	0.0002	0.77	0.0001	0.0002	0.61	0.0001	0.0005	0.90
Yogurt	0.0001	0.0006	0.98	0.0002	0.0007	0.81	-0.0007	0.0017	0.69
Cheese	0.0002	0.0003	0.65	0.0003	0.0003	0.45	-0.0012	0.0012	0.34
Milk + Yogurt	0.0001	0.0002	0.67	0.0001	0.0002	0.51	0.0000	0.0005	0.97
Milk + Yogurt + Cheese	0.0001	0.0002	0.53	0.0002	0.0002	0.32	-0.0001	0.0004	0.78

^**1**^Models adjusted for sex, cohort, height, weight, current smoking, energy intake, calcium supplement use, vitamin D supplement use, physical activity and multivitamin use

^**2**^Models for all participants were additionally adjusted for age

*SE* Standard error

In secondary analyses, when full models in the combined sample of men and women were stratified by age, no significant associations were observed (P range: 0.32–0.97, Table 3). Similarly, no significant associations were observed when the full models were stratified by both age and sex (i.e. females < 60 years, males < 60 years, females ≥ 60 years, and males ≥ 60 years; data not shown).

## Discussion

In this cross-sectional study, higher intake of cheese and cheese + milk + yogurt, were favorably associated with TBS in both men and women in crude models. However, associations became non-significant after adjusting for relevant confounders. Furthermore, no significant associations were observed in sex-combined or age-stratified analyses.

The only cross-sectional study that reported on dairy foods and TBS observed that higher milk intake was associated with higher TBS [least square adjusted means (SD) of TBS: < 1 glass/week (1.185 ± 0.084), several glasses/week (1.193 ± 0.077), 1 glass/day (1.198 ± 0.087), and 2 glasses/day or more (1.202 ± 0.082), P trend = 0.01] in 1,479 community dwelling older Japanese men [mean age 73.0 (SD 5.1) y] by Sato et al. [20]. However, we observed no significant associations with any of our select dairy foods and TBS in the FHS. Although it is not possible to directly compare the findings of the study by Sato et al. with those in the current study due to different measures and units used for estimating milk intake (i.e. dietary records to estimate glasses of milk per day in the study by Sato et al. versus FFQ to estimate servings of dairy per day in the FHS), it is important to note that in contrast to the study by Sato et al. [20], FHS included relatively younger men and women (mean age 49, SD:13 y) with relatively higher TBS, which could be a potential explanation for the null findings because older adults have significantly lower measures of TBS compared to the younger counterparts [31]. Additionally, FHS participants consume higher levels of milk intake (median intake: ~ 4 servings/day) compared to participants in the study by Sato et al. (median intake: 1 glass/day). Furthermore, a study that included 1,576 Australians with a wide age range (24–98 y) from the Geelong Osteoporosis study showed no association of dietary calcium and vitamin D (nutrients that are typically provided by dairy foods) with TBS [29], which is consistent with our findings. Thus, it is possible that intake of dairy food may be most useful for older adults that are prone to bone-loss.

A recent systematic review reported that there is moderate evidence for a role of dairy intake on bone health of older adults aged 50 years and above [12]. Most of the RCTs and all of the large cross-sectional studies included in the systematic review showed a beneficial impact of dairy foods on bone mineral content (BMC) or BMD. Previous studies have reported on the association of dairy foods, primarily milk intake with bone measures. The majority of these studies were focused on DXA-derived BMD rather than TBS [6, 32–39]. A cross-sectional study [34] showed that a higher dairy intake was associated with a greater hip aBMD in men ≥ 60 y, but not in women. In the Framingham Offspring Study, milk was associated with hip but not spine aBMD in men and women (mean age: 55 y) [6]. Furthermore, of the several dairy foods examined in this study only milk + yogurt + cheese intake was associated with higher L2-4 spine BMD [6]. However, no association was observed between dairy food intakes with either femur or spine aBMD in older men and women (aged 67–93 y) from the Framingham Original cohort, which is consistent with our results [36]. A cross-sectional analysis in postmenopausal (mean age 65 y) women reported that usual consumption of fermented dairy foods defined as at least 1 serving/week) was associated with higher lumbar spine BMD compared to non-consumers [17]. In the Study of Women's Health Across the Nation (SWAN), rate of bone loss at the femoral neck and lumbar spine over 10 years did not differ significantly by four dairy groups (dairy intake cumulatively averaged over the follow-up). Although these studies have linked dairy food intake with spinal BMD rather than TBS, the two measures appear to be highly correlated in previous studies [31] as well as in the current study. On average, BMD in the lumbar spine either remains stable or increases after age 60 y among women or after age 40 y among men, whereas BMD in the hips declines, except in peri-menopausal women whose bone loss in the lumbar spine is concordant with the pattern in the hips [40]. On the other hand, the magnitude of TBS decline between 45 and 85 y of age is reported to be ~ 14.5%. The rate of decline in TBS increases after 65 y by 50% [41].

Several studies have linked dairy foods with BMD at bone sites beyond the spine. A cross-sectional study of women aged 44–74 y reported that higher self-reported milk consumption before the age of 25 y was associated with higher BMD at total hip, femoral neck, trochanter, intertrochanteric region (*P* < 0.05 each), and Ward’s triangle (*P* < 0.005) [10]. Results from the Rancho Bernardo Study showed that regular milk consumption in youth was associated with higher BMD at cortical and trabecular sites in 581 white older women (mean age: 71 y) [11]. Relatively younger women aged 20–49 y from the third National Health and Nutrition Examination Survey, who consumed < 1 serving of milk per week during childhood (ages 5–12 y) were found to have 3% lower hip BMD (*P* < 0.02) than those who consumed > 1 serving per day [7].

Biver et al. [17] also reported that consumption of at least 2 serving per week of fermented dairy products was associated with attenuated loss of cortical bone at the radius after 3 y. Additionally, two other cross-sectional studies have focused on the associations between tibial/radial bone microarchitecture and dairy protein instead of dairy foods. Both studies have reported that higher dairy protein was associated with greater bone strength in older men [15] and post-menopausal women [16], yet no associations were seen with vegetable/plant protein [15, 16]. It is important to note that studies that found a favorable associations between fermented dairy products [17] or dairy protein and bone microarchitecture [15, 16], evaluated bone measures at the radius and/or the tibia of the extremities. Our study utilizes TBS that evaluates the bone texture at the base of the spine (lumbar vertebrae)—a different anatomical location. Thus, it is also possible that dairy food/diary protein intake may be more be more important for appendicular bones (e.g. bones in the arms and legs). More research is warranted to determine if the associations between dairy foods and bone texture is anatomically site-specific.

Considerable controversy remains given that other studies have reported favorable associations between dairy foods and spinal assessment of bone [17, 35]. It is difficult to draw an overall consensus of the association between dairy foods and spinal bone measures since there are several various evaluations of spinal bone. aBMD is an evaluation of bone mass and density, whereas TBS (an evaluation of bone texture) and HRp-QCT (an evaluation of bone microarchitecture) assess bone attributes beyond mass and density. Additionally, a meta-analysis of 6 randomized controlled trials found that greater dairy food intake prevented bone loss (as measured by BMD) over time [42]. Thus, it is possible that dairy food is most important for the prevention of bone loss, rather than bone texture or microarchitecture.

The strengths of this current study include use of a well-characterized cohort of community-dwelling adults with data on specific types of dairy foods. This study included TBS, a widely-used clinical estimate of bone texture. However, the limitations of TBS must also be noted. TBS is a texture analysis of bone, and does not provide detailed evaluation of bone strength or quality like other bone measures (e.g., HR-pQCT or BMD). Moreover, the TBS assessments appear to vary based on the anatomical site and amount of soft tissue surrounding the area, and do not always correlate well with bone microarchitecture [43, 44].

The limitations of this study include that our study population contained primarily men and women of European ancestry, which limits the generalizability of our study conclusions. We did not account for multiple comparisons in our analyses. It is also possible that there was misclassification of dietary intake due to recall bias that may occur with the use of a semi-quantitative FFQ. Furthermore, we did not account for the dietary patterns of participants, which may impact the absorption of key nutrients (e.g., calcium and vitamin D). Thus, future work should address the influence of dietary patterns on the association between dairy food and bone outcomes. Despite attempts to adjust for confounding, residual confounding likely remains. And finally, give then the cross-sectional nature of this study, we cannot infer causality.

## Conclusion

In conclusion, the results suggest that dairy food intake was not associated with TBS (an analysis of bone texture) in this cohort of primarily healthy adult men and women. Based on the previous literature supporting a role of dairy foods on bone mineral density, and our findings of no association with TBS, one could conclude that dairy foods are important for a uniform contribution to mineral content but not in a specific pattern to influence the texture of bone*.* Future studies should aim to confirm our findings on bone texture and dairy foods in other cohorts particularly in using longitudinal study design to improve the understanding of the role diet may play in bone health.

## Data Availability

Data described in the manuscript, code book, and analytic code will be made available upon request pending application to and approval by the Framingham Heart Study.
